# Supplementary material for: Movements and dispersal of brown trout (Salmo trutta Linnaeus, 1758) in Mediterranean streams: influence of habitat and biotic factors
Source: PeerJ. 2018 Oct 12;6:e5730. doi: 10.7717/peerj.5730 (PMC6188007; doi:10.7717/peerj.5730)
Supplement: Table S1 — FLM: Flamisell, NP: Noguera Pallaresa, and NV: Noguera Vallferrera. Season, Pre-Spawn and Post-Spawn models are shown. Model-averaged regression coefficients (β) are parameter coefficients averaged by model weight (wi) across all candidate models (ΔAICc < 2) in which the given parameter occurs; selection probability (SP) indicates the importance of an independent variable, and parameter bias is the difference between the averaged estimates ( β) and the full model coefficients. The number (N) of candidate models (ΔAICc < 2) and Pearson’s correlation coefficient (r) between observed and model predicted values are also shown. Parameters included in the best model, in each case, are highlighted in grey colour. [file peerj-06-5730-s001.docx]

| **Model parameter** | **Season Model**  ***N* = 9; *r* = 0.56** | | |  |
| --- | --- | --- | --- | --- |
|  | ***β*** | **SP** | **Bias** |  |
|  |  |  |  |  |
| Intercept | 61.416 |  | 0.443 |  |
| River - NP | -26.510 | 1.000 | -0.096 |  |
| River - NV | -2.814 | 1.000 | -0.280 |  |
| Fish Cover (%) | -1.067 | 1.000 | -0.166 |  |
| Fish Biomass (kg ha^-1^) | -0.016 | 0.694 | -1.420 |  |
| Substrate Coarseness | 3.329 | 0.424 | -1.178 |  |
| Organic Substrate (%) | 0.262 | 0.269 | 0.790 |  |
| Fish Density (Ind ha^-1^) | 0.010 | 0.162 | -4.182 |  |
| Water velocity (m s^-1^) | 0.335 | 0.069 | -1.409 |  |
| Mean Depth (cm) | *Not selected* | | |  |
| Pre Spawn Season | *Not selected* | | |  |
|  |  |  |  |  |
