# Supplementary figures and images for: Movements and dispersal of brown trout (Salmo trutta Linnaeus, 1758) in Mediterranean streams: influence of habitat and biotic factors
Source: PeerJ. 2018 Oct 12;6:e5730. doi: 10.7717/peerj.5730 (PMC6188007; doi:10.7717/peerj.5730)

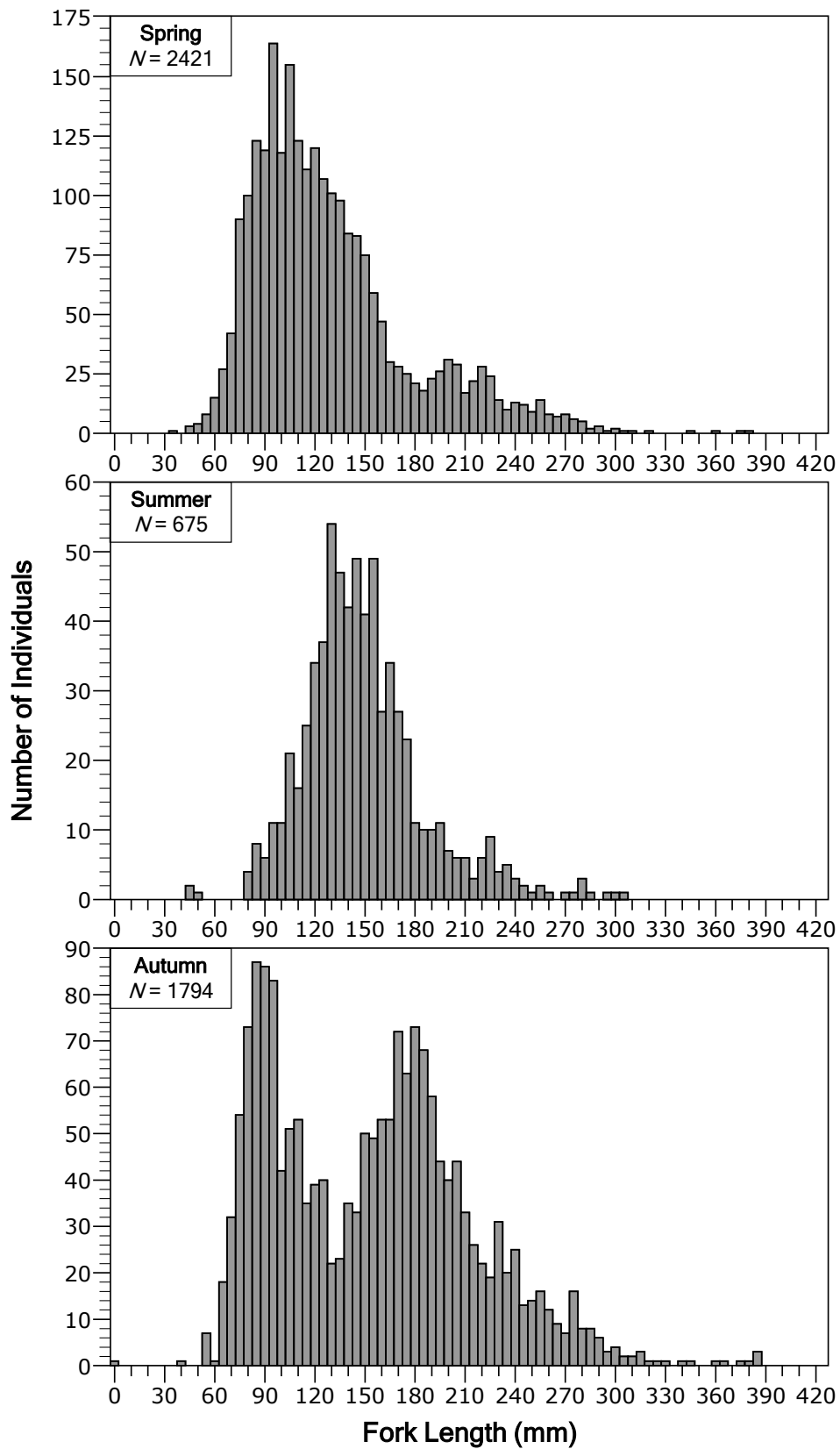

Supplement: Figure S1 [file peerj-06-5730-s002.pdf]

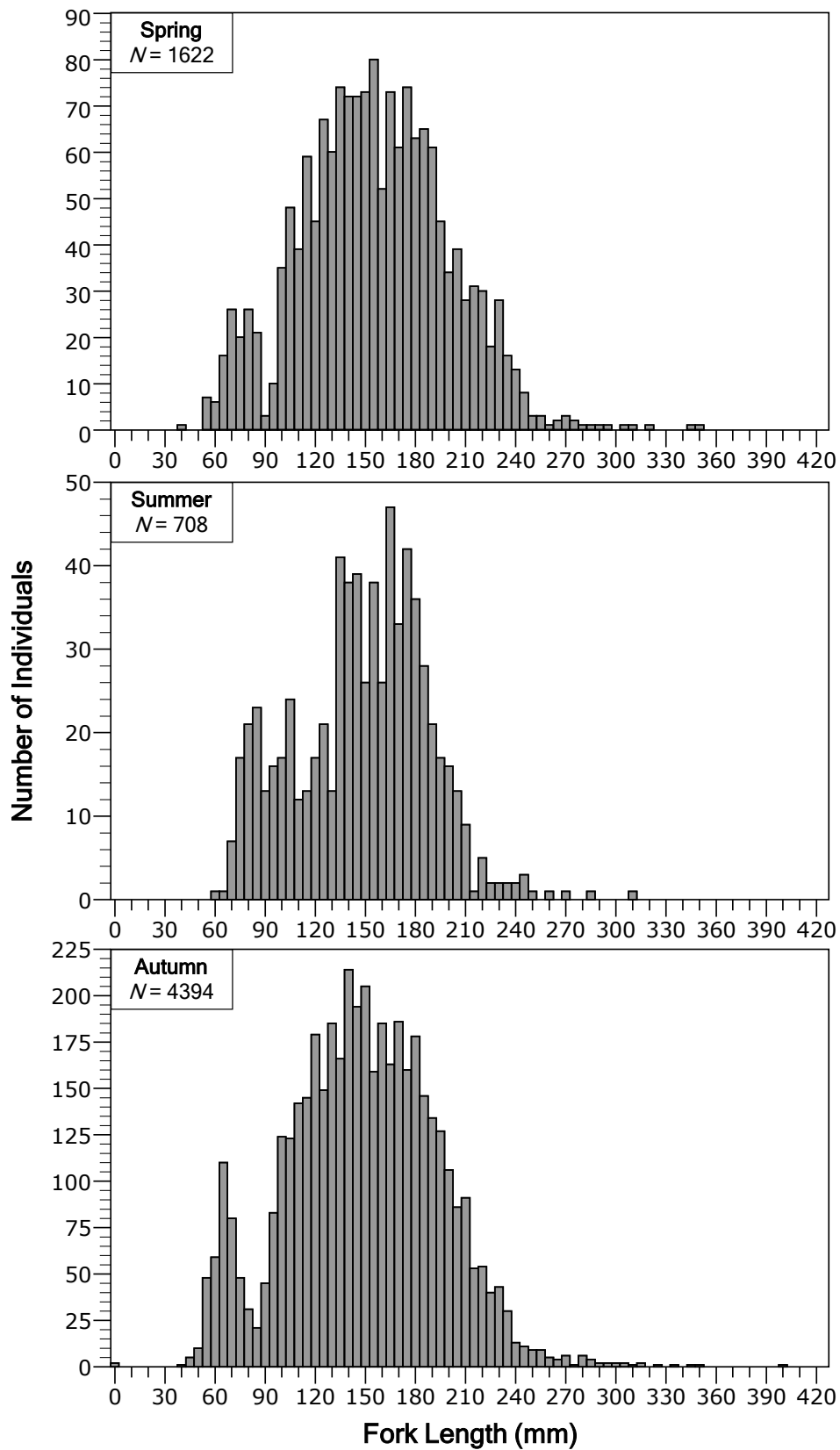

Supplement: Figure S2 [file peerj-06-5730-s003.pdf]

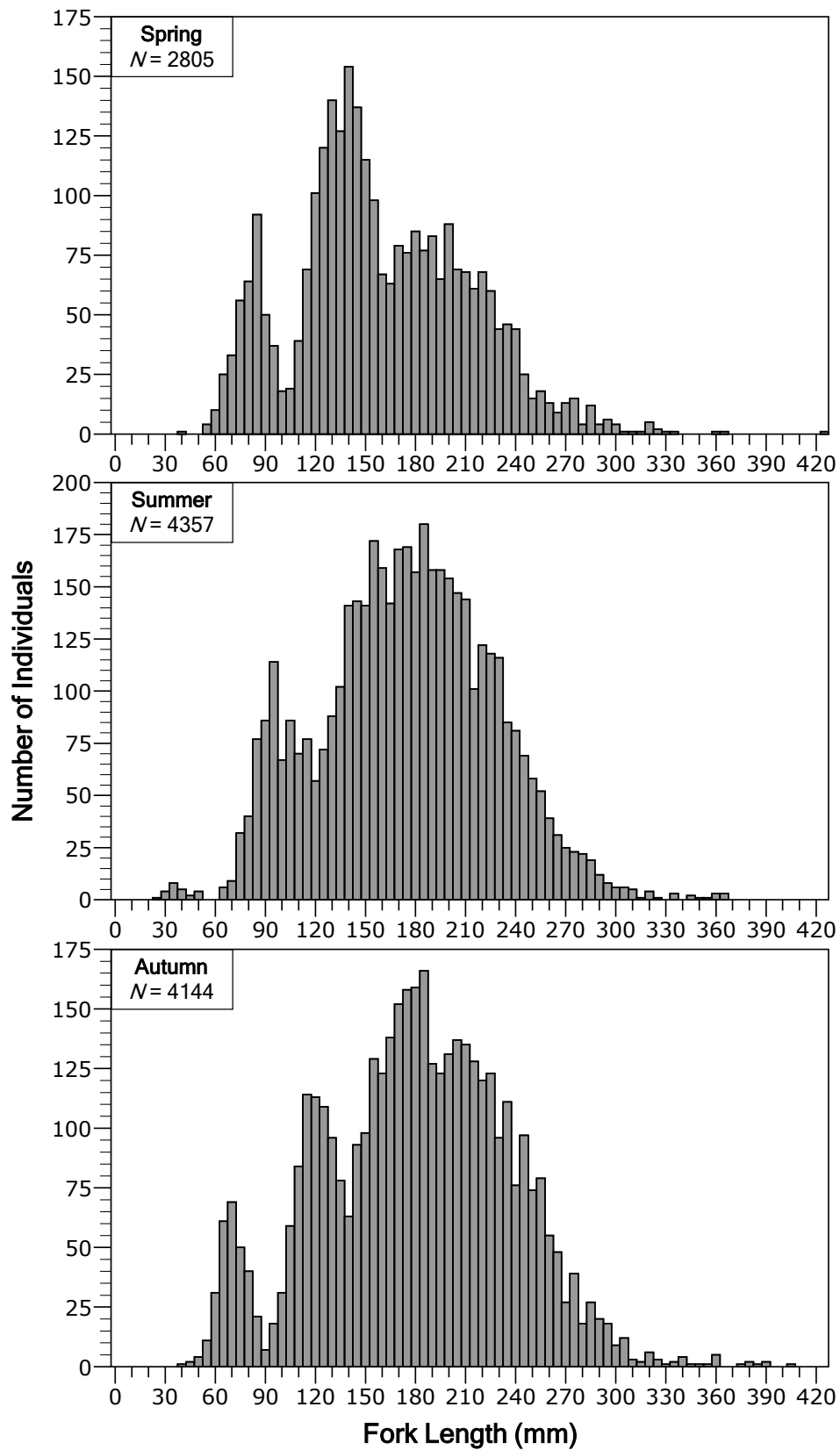

Supplement: Figure S3 [file peerj-06-5730-s004.pdf]
